# Remnant cholesterol is correlated with retinal vascular morphology and diabetic retinopathy in type 2 diabetes mellitus: a cross-sectional study

**DOI:** 10.1186/s12944-024-02064-6

**Published:** 2024-03-11

**Authors:** Shuli Chen, Yi Xu, Bo Chen, Senlin Lin, Lina Lu, Minna Cheng, Yuheng Wang, Qinping Yang, Saiguang Ling, Dengji Zhou, Yan Shi, Haidong Zou, Yingyan Ma

**Affiliations:** 1 Department of Eye Disease Control and Prevention, Shanghai Eye Disease Prevention & Treatment Center/Shanghai Eye Hospital, School of Medicine, Tongji University, No. 1440, Hongqiao Road, Shanghai, 200336 China; 2 Department of Ophthalmology, Shanghai General Hospital, Shanghai Jiao Tong University School of Medicine, No. 100, Haining Road, Shanghai, 200080 China; 3 National Clinical Research Center for Eye Diseases, Shanghai, China; 4 Shanghai Engineering Center for Precise Diagnosis and Treatment of Eye Diseases, Shanghai, China; 5 Department of Chronic Non-Communicable Diseases and Injury, Shanghai Municipal Center for Disease Control & Prevention, No. 1380, West Zhongshan Road, Shanghai, China; 6 School of Public Health, Fudan University, No. 130, Dongan Road, Shanghai, China; 7 EVision technology (Beijing) co. LTD, Beijing, 100085 China; 8 National Clinical Research Center for Aging and Medicine, Huashan Hospital, Fudan University, No. 12, Middle Wulumuqi Road, Shanghai, China

**Keywords:** Diabetic retinopathy, Remnant cholesterol, Retinal vessels, Type 2 diabetes mellitus

## Abstract

**Background:**

The association between remnant cholesterol (RC) and diabetic retinopathy (DR) in type 2 diabetes mellitus (T2DM) remains unclear. Morphological changes in retinal vessels have been reported to predict vascular complications of diabetes, including DR.

**Methods:**

This cross-sectional study included 6535 individuals with T2DM. The RC value was calculated using the recognized formula. The retinal vascular parameters were measured using fundus photography. The independent relationship between RC and DR was analyzed using binary logistic regression models. Multiple linear regression and subgroup analyses were employed to investigate the link between RC and vascular parameters, including the retinal arteriolar diameter (CRAE), venular diameter (CRVE), and fractal dimension (D_f_). Mediation analysis was performed to assess whether the vascular morphology could explain the association between RC and DR.

**Results:**

RC was independently associated with DR in patients with a longer duration of T2DM (> 7 years). Patients with the highest quartile RC levels had larger CRAE (5.559 [4.093, 7.025] μm), CRVE (7.620 [5.298, 9.941] μm) and D_f_ (0.013 [0.009, 0.017]) compared with patients with the lowest quartile RC levels. Results were robust across different subgroups. The association between RC and DR was mediated by CRVE (0.020 ± 0.005; 95% confidence interval: 0.012–0.032).

**Conclusions:**

RC may be a risk factor for DR among those who have had T2DM for a longer period of time. Higher RC levels were correlated with wider retinal arterioles and venules as well as higher D_f_, and it may contribute to DR through the dilation of retinal venules.

**Supplementary Information:**

The online version contains supplementary material available at 10.1186/s12944-024-02064-6.

## Background

Diabetes is a prevalent and significant chronic disease in contemporary society, the global prevalence of which is projected to reach 12.2% (783.2 million individuals) by 2045 [1]. The high morbidity and disability associated with diabetes have emerged as pressing public health concerns, posing a significant threat to human well-being and the global economy [2]. Diabetic retinopathy (DR) is a major contributor to diabetes-related disability [3]. However, known risk factors fail to explain the significant individual variations in the development and severity of DR, and the identification of additional risk factors for disease control is therefore required [4].

Dyslipidemia is a characteristic metabolic disorder in diabetes, typically manifesting at the early stages and hastening the onset of diabetic complications, including DR [5–7]. The role of remnant cholesterol (RC) has recently attracted growing attention. RC represents the cholesterol content in triglyceride-rich lipoproteins (TRLs), including very low- and intermediate-density lipoproteins as well as chylomicron remnants [8]. Large epidemiological studies have substantiated the close association between RC and the occurrence, as well as the progression of cardiovascular diseases (CVDs) in both individuals with and without diabetes, with a potential role in promoting atherosclerosis potentially surpassing that of low-density lipoprotein cholesterol (LDL-C) [9–14]. Unlike the well-established causal relationship between RC and CVDs, the connection between RC and microvascular disease remains uncertain. According to a multicenter cohort study conducted in Finland, the RC concentration was predictive of diabetic nephropathy and severe DR in patients with type 1 diabetes mellitus (T1DM) [15]. For those with type 2 diabetes mellitus (T2DM), a study in China revealed a positive association between RC and DR prevalence [16]. However, another cross-sectional study reported no significant correlation between the two [17]. To date, the relationship between RC and DR in the population with T2DM remains inconclusive.

This study attempted to clarify the impact of RC on DR by investigating the association between RC and retinal vascular morphology. The retinal microvasculature is the only directly observable deep microvascular system in the human body [18]. With the development of fundus photography technology and associated analysis software, the visuality and quantifiability of the structural patterns of the retinal microvasculature have enabled it to become a reliable research indicator [19]. Previous studies demonstrated that the retinal vascular diameter and geometric parameters (e.g., vascular tortuosity [VT], fractal dimension [D_f_]) are related to the risk of ocular and systemic diseases, such as DR, diabetic nephropathy, ischemic stroke, coronary heart disease, hypertension, and other cardio- and cerebrovascular diseases [20–24]. Structural and functional alterations in retinal blood vessels can predict micro- and macrovascular complications of diabetes, thereby facilitating early recognition of DR at a preclinical stage [25]. It is well accepted that the early morphological changes in retinal vessels in the context of diabetes are attributed to elevated blood glucose levels and the secondary persistent hypoxia and chronic inflammation [26–29]. However, minimal attention has been paid to the impact of abnormal metabolism of lipids (such as RC) on retinal vascular morphology.

This study targeted adult patients with T2DM, aiming to explore the associations between RC and retinal microvascular morphology, as well as DR, and to explore whether the connection between RC and DR is mediated by retinal microvascular morphology.

## Methods

### Participants

Patients were sourced from the Shanghai Cohort Study of Diabetic Eye Disease (SCODE). The inclusion and exclusion criteria for participants and the diagnostic criteria of T2DM and DR employed in the SCODE were previously described [30, 31]. According to the Declaration of Helsinki, this study was approved by the Ethics Committee of Shanghai General Hospital. Written informed consent was obtained from each participant.

### Data collection

The basic information of patients was documented, including gender, date of birth, body mass index(BMI), blood pressure, time of diagnosis, and medical history. The mean arterial pressure (MAP) was computed as 1/3 of systolic blood pressure (SBP) plus 2/3 of diastolic blood pressure (DBP). Blood samples were collected for testing indicators of blood glucose (glycated hemoglobin A1c [HbA1c] and fasting plasma glucose [FPG]) and lipids (total cholesterol [TC], triglycerides [TG], high-density lipoprotein cholesterol [HDL-C], and LDL-C). RC = TC − HDL-C − LDL-C. Participants underwent routine ophthalmic examinations. Digital fundus photography (Topcon NW400; Topcon, Tokyo, Japan) was performed with or without mydriasis, and colored photographs centered on macula and optic disc were taken at a 45° angle for each eye.

### Retinal vascular parameters

Optic disc-centered photos of the right eye were used for analyses; in cases where these were unavailable, photos of the left eye were used instead.

**Fig. 1:**
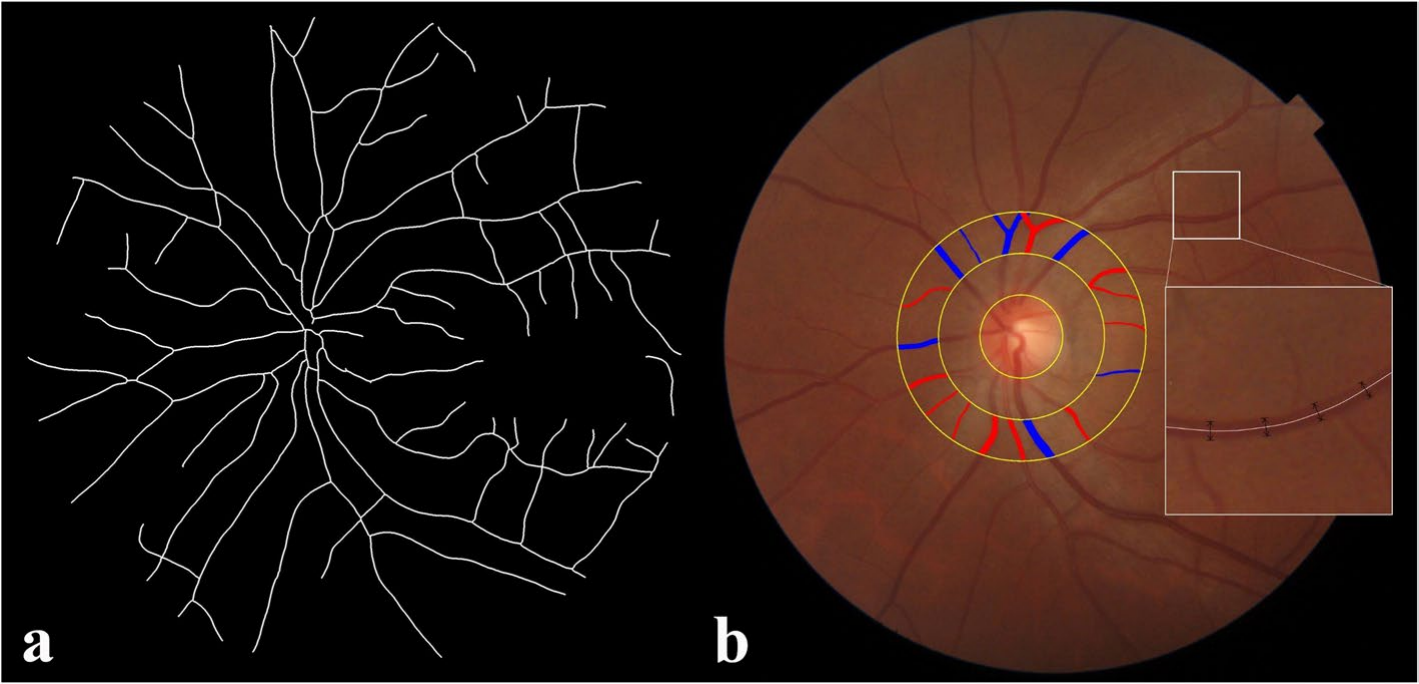
Illustrations for analyses of retinal vascular parameters. **a** displayed the preprocessed fundus image. **b** depicted the retinal arterioles (red line) and venules (blue line) within 0.5–1 PD from the optic disc edge and the way to measure the vascular diameters

Computer vision and deep learning technology based on the bionic mechanism of human vision were integrated to automatically measure the vessel diameters, VT, and D_f_ from retinal photographs. First, the fundus image was preprocessed, followed by extraction of the region of interest to obtain the target area. Subsequently, denoising, normalization and image enhancement were performed to enhance the significance of blood vessels (Fig. 1a) [32]. The preprocessed fundus image was fed into ResNet101-UNet for vascular segmentation. The segmented blood vessels were subjected to morphological erosion to extract their centerlines. A line perpendicular to a specific point on the centerline intersects the vascular edges at two points, the distance between which represents the vascular diameter corresponding to that specific point (Fig. 1b). Then, a deep learning network and an edge extraction algorithm were synergistically employed for precise localization of the optic disc. The papillary diameter (PD) was determined according to the smallest external circle within the segmentation area, and the diameter of all blood vessels within 0.5–1 PD from the optic disc edge was calculated. The six arteriolar and six venular vessels with the largest average diameters were selected, and the central retinal artery equivalent (CRAE) and central retinal vein equivalent (CRVE) were calculated according to established methodologies [33]. The ratio of CRAE to CRVE was denoted as arteriole-to-venule ratio (AVR). The measurement of VT and D_f_ has been described elsewhere [34, 35].

### Statistical analysis

Continuous variables were reported as mean ± standard if distributed normally, or as median (inter-quartile range) if not. Differences between multiple independent groups were compared using ANOVA or the Kruskal–Wallis H test. Categorical variables were reported as frequency (percentage), and the chi-square test was employed to compare the proportions. According to the quartile, RC was converted into a categorical variable.

To investigate the relationship between RC and DR, the participants were stratified by the median duration of T2DM (≤ 7 years and > 7 years), and four distinct logistic regression models were constructed: crude model (without adjusting for any covariates), Model 1 (adjusting for age and gender), Model 2 (further adjusting for BMI, T2DM duration, HbA1c, and MAP) and Model 3 (further adjusting for TG, HDL-C, and LDL-C). The association between the vascular parameters and DR was also examined using the models. Multiple linear regression with four different models (as previously described) was conducted to explore the correlation between RC and vascular parameters. Participants were grouped based on age, gender, T2DM duration, BMI, HbA1c, MAP, and DR prevalence, allowing further investigation of the relationship between RC and vascular morphological parameters in each subgroup. The *P*-trend was calculated based on RC as a continuous variable containing the median of each quartile.

Multiple parallel mediation analyses adjusting for age, gender, BMI, T2DM duration, HbA1c, MAP, TG, HDL-C, and LDL-C were performed to quantify the extent to which the association between RC and DR was mediated by each retinal vascular morphological parameter. Bootstrapping with 5000 resamples was used to verify the indirect effects, where 95% confidence intervals (CIs) that did not cross zero were considered significant.

Data analysis was conducted with SPSS 26.0 and Mplus 8.3 software. *P* < 0.05 (two-tailed) was deemed statistically significant.

## Results

A total of 6627 patients were initially enrolled, 67 and 25 of whom were excluded due to missing data and RC outliers, respectively. Consequently, 6535 patients were incorporated into the final analysis. The characteristics of the participants stratified by RC-level quartiles were summarized in Table 1. Individuals with higher RC levels were more likely to be women and younger individuals and to have had a shorter course of diabetes, lower HDL-C, and higher BMI, FPG, MAP, TC, and TG. The patients were then stratified according to median T2DM duration. Among those with a course > 7 years, the prevalence of DR significantly differed among the four groups. In terms of the retinal vascular morphological parameters, CRAE, CRVE, and D_f_ were significantly higher in individuals with higher RC levels. Additionally, AVR and VT varied significantly among the different RC groups (Table 1).

**Table 1:** Characteristics of the patients stratified by RC-level quartiles

RC Quartile	Q1	Q2	Q3	Q4	All	*P* value
n	1647 (≤ 0.35)	1622 (0.36 − 0.58)	1663 (0.59 − 0.90)	1603 (0.91 − 10.89)	6535	
T2DM duration ≤ 7 years	856	899	909	911	3575	
T2DM duration > 7 years	791	723	754	692	2960	
RC (mmol/L)	0.20 (0.10 − 0.30)	0.47 (0.41 − 0.52)	0.72 (0.65 − 0.80)	1.23 (1.03 − 1.61)	0.58 (0.35 − 0.90)	< 0.001
Age (years)	65 (61 − 70)	64 (60 − 69)	64 (60 − 68)	64 (59 − 68)	64 (60 − 69)	< 0.001
Male, n (%)	781 (47.4%)	714 (44.0%)	663 (39.9%)	675 (42.1%)	2833 (43.4%)	< 0.001
T2DM duration (years)	7 (4 − 12)	7 (4 − 11)	7 (4 − 12)	6 (3 − 11)	7 (4 − 11)	< 0.001
BMI (kg/m^2^)	24.09 (22.23 − 26.08)	24.22 (22.48 − 26.26)	24.54 (22.94 − 26.84)	24.88 (23.01 − 26.90)	24.44 (22.65 − 26.56)	< 0.001
FPG (mmol/L)	7.2 (6.3 − 8.3)	7.3 (6.4 − 8.5)	7.3 (6.4 − 8.7)	7.5 (6.5 − 9.0)	7.3 (6.4 − 8.6)	< 0.001
HbA1c (%)	7.4 (6.5 − 7.8)	7.1 (6.4 − 7.8)	7.2 (6.4 − 7.9)	7.2 (6.6 − 8.0)	7.2 (6.5 − 7.9)	0.028
SBP (mmHg)	130 (124 − 134)	130 (122 − 133)	130 (123 − 134)	130 (122 − 134)	130 (122 − 134)	0.242
DBP (mmHg)	78 (74 − 80)	80 (74 − 82)	78 (74 − 82)	80 (75 − 82)	78 (74 − 82)	< 0.001
MAP (mmHg)	94.7 (91.7 − 97.3)	95.0 (92.0 − 98.7)	95.0 (92.0 − 98.3)	95.3 (92.0 − 98.7)	95.0 (92.0 − 98.0)	< 0.001
Hypertension, n (%)	1058 (64.2%)	1018 (62.8%)	1118 (67.2%)	1100 (68.6%)	4294 (65.7%)	0.001
TC (mmol/L)	4.43 (3.81 − 5.06)	4.60 (4.02 − 5.21)	4.90 (4.28 − 5.55)	5.24 (4.58 − 5.99)	4.79 (4.15 − 5.47)	< 0.001
TG (mmol/L)	1.10 (0.80 − 1.50)	1.10 (0.90 − 1.50)	1.60 (1.40 − 1.90)	2.50 (2.10 − 3.60)	1.50 (1.10 − 2.20)	< 0.001
HDL-C (mmol/L)	1.40 (1.21 − 1.71)	1.40 (1.18 − 1.65)	1.31 (1.11 − 1.54)	1.14 (0.97 − 1.34)	1.31 (1.10 − 1.57)	< 0.001
LDL-C (mmol/L)	2.68 (2.08 − 3.33)	2.67 (2.15 − 3.21)	2.81 (2.24 − 3.39)	2.67 (2.09 − 3.30)	2.70 (2.14 − 3.30)	< 0.001
DR, n (%)	201 (12.2%)	217 (13.4%)	213 (12.8%)	223 (13.9%)	854 (13.1%)	0.508
T2DM duration ≤ 7 years	83 (9.7%)	71 (7.9%)	77 (8.5%)	87 (9.5%)	318 (8.9%)	0.482
T2DM duration > 7 years	118 (14.9%)	146 (20.2%)	136 (18.0%)	136 (19.7%)	536 (18.1%)	0.034
NPDR, n (%)	197 (12.0%)	212 (13.1%)	204 (12.3%)	221 (13.8%)	834 (12.8%)	0.397
PDR, n (%)	4 (0.2%)	5 (0.3%)	9 (0.5%)	2 (0.1%)	20 (0.3%)	0.179
Insulin use, n (%)	151 (9.2%)	157 (9.7%)	156 (9.4%)	127 (7.9%)	591 (9.0%)	0.320
CRAE (μm)	137.77 (127.37 − 147.81)	138.65 (129.33 − 148.54)	138.50 (129.84 − 148.07)	138.71 (129.59 − 149.16)	138.39 (128.99 − 148.49)	0.004
CRVE (μm)	228.49 (211.60 − 244.63)	229.13 (213.62 − 245.51)	231.99 (214.94 − 247.21)	232.28 (215.36 − 250.36)	230.31 (213.82 − 246.84)	< 0.001
AVR	0.60 (0.56 − 0.65)	0.61 (0.56 − 0.65)	0.60 (0.56 − 0.65)	0.60 (0.56 − 0.65)	0.60 (0.56 − 0.65)	0.037
VT, × 10^–4^	9.40 (8.23 − 10.61)	9.36 (8.04 − 10.45)	9.41 (8.24 − 10.51)	9.48 (8.34 − 10.62)	9.41 (8.23 − 10.53)	0.033
D_f_	1.48 (1.45 − 1.52)	1.50 (1.46 − 1.52)	1.50 (1.46 − 1.53)	1.50 (1.46 − 1.53)	1.49 (1.46 − 1.53)	< 0.001

### Relationship between RC and DR prevalence

No significant correlation between RC and DR was found among individuals with a T2DM duration of ≤ 7 years (Table 2). However, when the T2DM duration was > 7 years, RC could be a risk factor for DR. DR prevalence in Q2 and Q4 in all models and in Q3 in Model 3 was significantly higher than those in Q1. (Table 2).

**Table 2:** Binary logistic regression analyses for correlation between RC and DR

Variable	Crude model	Model 1	Model 2	Model 3
T2DM duration ≤ 7 years				
RC (Quartile)				
Q1	Ref	Ref	Ref	Ref
Q2	0.799 (0.573, 1.113)	0.809 (0.580, 1.127)	0.825 (0.591, 1.151)	0.813 (0.581, 1.137)
Q3	0.862 (0.623, 1.193)	0.881 (0.636, 1.221)	0.878 (0.632, 1.220)	0.847 (0.607, 1.183)
Q4	0.983 (0.717, 1.349)	1.009 (0.734, 1.387)	1.013 (0.735, 1.396)	0.965 (0.667, 1.397)
*P*-trend	0.790	0.671	0.687	0.983
T2DM duration > 7 years				
RC (Quartile)				
Q1	Ref	Ref	Ref	Ref
Q2	1.443 (1.105, 1.885) **	1.432 (1.096, 1.871) **	1.467 (1.117, 1.926) **	1.464 (1.114, 1.924) **
Q3	1.255 (0.958, 1.644)	1.242 (0.947, 1.629)	1.276 (0.968, 1.683)	1.345 (1.013, 1.786) *
Q4	1.395 (1.064, 1.829) *	1.378 (1.049, 1.810) *	1.427 (1.079, 1.888) *	1.868 (1.339, 2.607) ***
*P*-trend	0.061	0.078	0.051	0.001

Data were presented as odds ratio (95% CI). *P < 0.05, **P < 0.01, ***P < 0.001. Ref., reference

Crude model: unadjusted

Model 1: age and gender

Model 2: Model 1 + BMI, T2DM duration, HbA1c, and MAP

Model 3: Model 2 + TG, HDL-C, and LDL-C

### Correlation between RC and retinal vascular morphological parameters

The effect of RC on retinal vascular morphological parameters was analyzed using a linear regression model (Table 3). In the crude model and Models 1, 2, and 3, the CRAE and D_f_ of the participants in Q2, Q3, and Q4 and the CRVE of the participants in Q3 and Q4, were significantly higher than those in Q1. In the final model, the CRAE, CRVE, and D_f_ exhibited an average increase of 5.559 (4.093, 7.025) μm, 7.620 (5.298, 9.941) μm, and 0.013 (0.009, 0.017), respectively, when comparing Q4 with Q1. There was no discernible linear correlation between RC and AVR or VT.

**Table 3:** Multiple linear regression analyses for correlation between RC and retinal vasculature

Variable	Crude model	Model 1	Model 2	Model 3
CRAE				
RC (Quartile)				
Q1	Ref	Ref	Ref	Ref
Q2	2.082 (0.814, 3.349) **	1.781 (0.519, 3.044) **	1.981 (0.721, 3.242) **	2.121 (0.861, 3.380) ***
Q3	2.588 (1.328, 3.847) ***	2.156 (0.898, 3.414) **	2.477 (1.219, 3.735) ***	3.044 (1.768, 4.320) ***
Q4	3.408 (2.137, 4.679) ***	2.861 (1.591, 4.132) ***	3.313 (2.039, 4.587) ***	5.559 (4.093, 7.025) ***
*P*-trend	< 0.001	< 0.001	< 0.001	< 0.001
CRVE				
RC (Quartile)				
Q1	Ref	Ref	Ref	Ref
Q2	1.402 (− 0.610, 3.414)	0.901 (− 1.092, 2.894)	1.011 (− 0.984, 3.006)	1.195 (− 0.799, 3.190)
Q3	3.533 (1.534, 5.533) ***	2.921 (0.935, 4.906) **	2.973 (0.981, 4.964) **	3.429 (1.409, 5.448) ***
Q4	6.001 (3.983, 8.019) ***	5.054 (3.048, 7.060) ***	5.113 (3.095, 7.131) ***	7.620 (5.298, 9.941) ***
*P*-trend	< 0.001	< 0.001	< 0.001	< 0.001
AVR				
RC (Quartile)				
Q1	Ref	Ref	Ref	Ref
Q2	0.006 (0.001, 0.011) *	0.006 (0.001, 0.011) *	0.007 (0.002, 0.012) **	0.007 (0.002, 0.012) **
Q3	0.003 (− 0.002, 0.007)	0.002 (− 0.003, 0.007)	0.004 (− 0.001, 0.009)	0.005 (− 0.000, 0.010)
Q4	0.000 (− 0.005, 0.005)	0.000 (− 0.005, 0.005)	0.002 (− 0.003, 0.007)	0.005 (− 0.000, 0.011)
*P*-trend	0.491	0.530	0.925	0.143
VT, × 10^–4^				
RC (Quartile)				
Q1	Ref	Ref	Ref	Ref
Q2	− 0.118 (− 0.246, 0.011)	− 0.119 (− 0.248, 0.009)	− 0.130 (− 0.259, -0.001) *	− 0.128 (− 0.257, 0.001)
Q3	− 0.012 (− 0.139, 0.116)	− 0.016 (− 0.144, 0.113)	− 0.037 (− 0.166, 0.091)	− 0.044 (− 0.175, 0.086)
Q4	0.063 (− 0.066, 0.192)	0.060 (− 0.069, 0.190)	0.031 (− 0.100, 0.161)	− 0.029 (− 0.180, 0.121)
*P*-trend	0.108	0.119	0.274	0.973
D_f_				
RC (Quartile)				
Q1	Ref	Ref	Ref	Ref
Q2	0.011 (0.007, 0.015) ***	0.008 (0.005, 0.011) ***	0.008 (0.005, 0.011) ***	0.008 (0.005, 0.012) ***
Q3	0.013 (0.009, 0.017) ***	0.009 (0.006, 0.013) ***	0.009 (0.006, 0.013) ***	0.011 (0.007, 0.014) ***
Q4	0.015 (0.011, 0.018) ***	0.009 (0.006, 0.013) ***	0.010 (0.006, 0.013) ***	0.013 (0.009, 0.017) ***
*P*-trend	< 0.001	< 0.001	< 0.001	< 0.001

Data were presented as coefficient (95% CI). *P < 0.05, **P < 0.01, ***P < 0.001. Ref., reference

Crude model: unadjusted

Model 1: age and gender

Model 2: Model 1 + BMI, T2DM duration, HbA1c, and MAP

Model 3: Model 2 + TG, HDL-C, and LDL-C

### Subgroup analyses for the relationship between RC and retinal vascular morphological parameters

CRAE, CRVE, and D_f_ were positively correlated with RC levels in distinct subgroups stratified by gender, age, T2DM duration, BMI, HbA1c, MAP, and DR status. This relationship was significant in most subgroups (Fig. S1, Fig. S2, and Fig. S3).

### Association between retinal vascular morphology and DR

In all binary logistic regression models, the five vascular parameters were significantly associated with DR. Specifically, CRAE, CRVE, and VT were positively correlated with DR, whereas AVR and D_f_ were negatively correlated with DR (Table S1).

### Retinal vascular morphology mediates the relationship between RC and DR

**Fig. 2:**
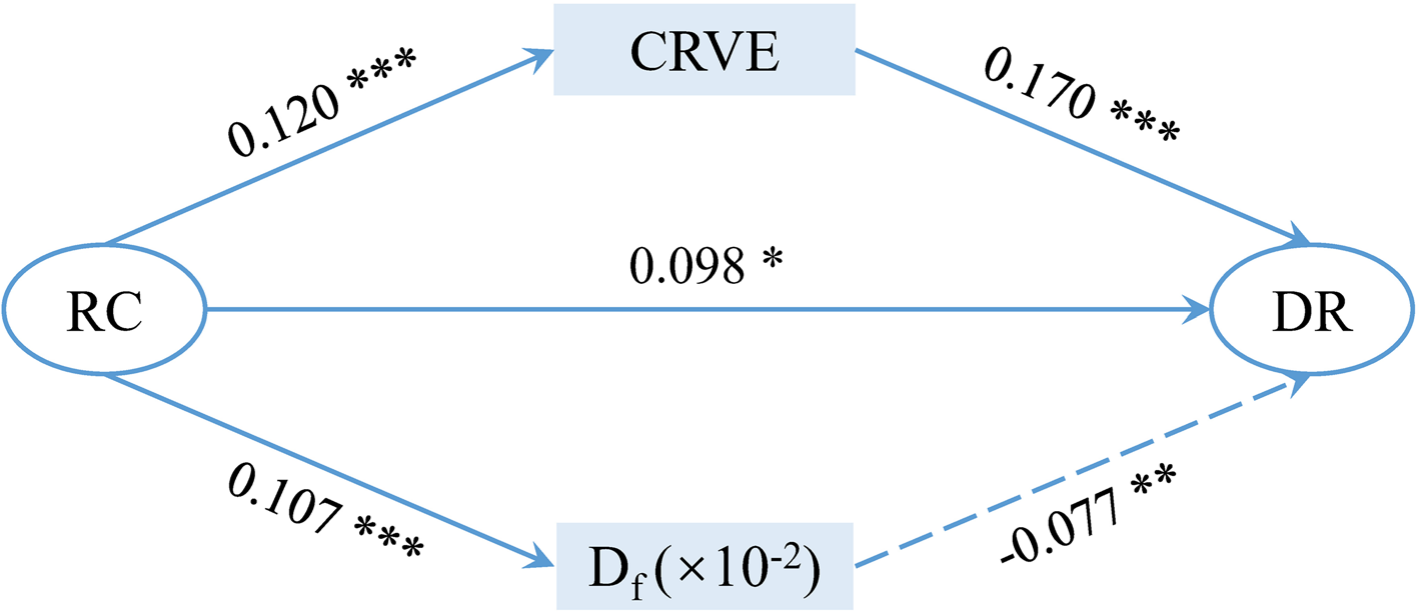
Mediators of the relationship between RC and DR. Only statistically significant paths were shown. Standardized coefficient for each path was presented. *P < 0.05, **P < 0.01, ***P < 0.001

Parallel mediation analysis was performed in participants with a duration of more than 7 years, including RC as the independent variable, DR as the dependent variable, and CRAE, CRVE, and D_f_ as mediators (Fig. 2, Table 4). CRAE had no significant mediating effect on the relationship between RC and DR (0.003; 95% CI [−0.003, 0.011]). CRVE was a significant mediator of this association (0.020; 95% CI [0.012, 0.032]). Conversely, D_f_ was a suppressor of this association (− 0.008; 95% CI [− 0.017, − 0.003]), but the suppressive effect was less pronounced than the mediating effect of CRVE. Therefore, the association between RC and DR is likely mediated by the diameter of the retinal venules.

**Table 4:** Standardized effects of the parallel mediation model

Indirect path	Estimate (S.E.)	95% CI		*Z* value	*P* value
		**Lower**	**Upper**		
CRAE	0.003 (0.003)	− 0.003	0.011	0.950	0.342
CRVE	0.020 (0.005)	0.012	0.032	3.961	< 0.001
D_f_, × 10^–2^	− 0.008 (0.004)	− 0.017	− 0.003	− 2.352	0.019

Estimate represented the mediating effect value, calculated by multiplying the standardized coefficients of the two paths before and after the mediators. 95% CI was obtained by bootstrapping. *Z* value and *P* value were obtained by Sobel test

## Discussion

The findings suggested that RC might be a risk factor for DR among individuals with T2DM with a duration of > 7 years. The retinal vascular morphological parameters, including CRAE, CRVE, and D_f_, were positively associated with RC in patients with T2DM. These vascular parameters also showed a significant correlation with DR. Moreover, the relationship between RC and DR may be mediated by the diameter of retinal venules (CRVE).

Few clinical studies have focused on the relationship between RC and DR against the background of T2DM. Only two cross-sectional studies have been conducted in China, and results were contradictory. In a study of 456 individuals in Harbin, Shan et al. discovered a positive correlation between RC levels and the prevalence and severity of DR [16]. Conversely, another study targeting 1956 participants in southern Taiwan found no significant association between RC and DR or proliferative DR (PDR) [17]. Herein, stratification based on T2DM duration revealed that higher levels of RC in patients with longer disease duration (> 7 years) were associated with a higher risk of DR. This implies that the influence of RC on the pathogenesis of DR may be subtle and slow, becoming more apparent as the disease progresses. Although substantial disagreements persist among the limited research findings, the potential impact of RC on DR cannot be ignored. Therefore, conducting more comprehensive longitudinal studies is imperative to prevent DR prevention in patients with T2DM.

Morphological and structural changes in the retinal microvascular system are considered preclinical signs of vascular complications of diabetes, including DR. In this study, five retinal vascular parameters were examined, among which AVR and VT showed no significant association with RC. Therefore, subsequent analyses focused on the other three vascular parameters: CRAE, CRVE, and D_f_. The two diameter parameters, CRAE and CRVE, were found to positively correlate with both RC and DR. However, the relationship between DR and CRAE, which characterizes the diameter of the retinal arterioles, remains controversial. Several prospective studies have shown that larger retinal arteriolar diameters can predict DR progression. Conversely, in young patients with T1DM in Denmark, a narrower retinal arteriolar diameter was associated with the onset of PDR [28, 36]. This divergence may be due to age, race, type of diabetes, or other factors, indicating the intricate nature of arteriole diameter regulation. In addition, a recent study found that owing to the central light reflex, CRAE could be overestimated in fundus camera images [37]. This measurement bias might partially explain the discrepancies reported in these studies. The association between DR and CRVE, which represents the diameter of the retinal venules, seemed clearer. Most studies demonstrated that a wider retinal venule diameter is associated with DR [26, 36, 38]. After stratification by gender, age, duration, BMI, HbA1c, MAP, and DR prevalence, a significant correlation remained between CRVE and RC in each subgroup, suggesting a strong association. Mediation analysis indicated that RC may contribute to the development of DR by inducing dilation of retinal venules. This finding emphasizes the role of alterations in retinal vascular morphology, particularly CRVE, in the pathological effects of RC on DR.

The parameter D_f_ quantifies the geometric complexity of the retinal vascular branching patterns [39]. Limited research has indicated that D_f_ may share a nonlinear relationship with the severity of DR, with an observed increase in D_f_ among patients with mild nonproliferative DR (NPDR) and a decrease among those with moderate to severe NPDR [40]. Patients who progressed to PDR exhibited a lower D_f_ than those who did not [26]. The increase in D_f_ during the early stages of DR may be attributed to increased arteriovenous shunting induced by ischemia and hypoxia. With the loss of peripheral cells and appearance of avascular zones, a decline in D_f_ may represent progression towards a more advanced stage of the disease. Herein, D_f_ increased significantly with increasing RC levels, which may have resulted from the predominance of non-DR participants (86.9%). Subsequently, a subgroup analysis was performed based on the presence or absence of DR and showed that the positive correlation between D_f_ and RC was no longer significant in the DR group. Mediation analysis and logistic regression indicated a negative association between D_f_ and DR, which may be related to the specific type of DR in the participants.

TGs in TRLs are hydrolyzed in the circulation, generating residual particles rich in cholesterol esters, the cholesterol part of which is called RC [41]. These residual particles are sufficiently small enough to freely pass through the vascular endothelial barrier and reside in the connective tissue matrix, thereby facilitating ample interactions with the vascular wall structure [42]. Although RC is famous for promoting atherosclerosis, alterations in the microvasculature are unrelated to this characteristic. A large clinical study reported a substantial causal relationship between RC and systemic inflammation [43]. The RC particles that accumulate in the arterial wall may elicit inflammation, thereby inducing vascular endothelial damage [44]. RC may trigger inflammation and endothelial dysfunction, causing the expansion of the retinal arterioles and venules. Conversely, RC may be also involved in promoting retinal ischemia and hypoxia under high glucose conditions, leading to more arteriovenous shunts (manifested as an increase in D_f_) and compensatory vasodilation in response to chronic hypoxia, thereby accelerating the onset of early DR [45, 46]. Currently, robust basic research evidence regarding this issue is scarce. A more comprehensive and precise mechanism warrants further exploration to provide in-depth insight into the role of RC in microvascular diseases.

## Study strengths and limitations

The present study elucidated the association between RC and retinal vascular morphology for the first time, and the findings revealed the intermediate role of morphological alterations in retinal vessels in DR induced by RC. Moreover, the large sample size enhances the validity of the results. However, the study has certain limitations. First, the RC value obtained indirectly through the calculation formula may lack precision compared with the direct measurement. Second, this study only examined a limited range of vascular parameters, thus failing to provide a comprehensive depiction of the morphological alterations in the fundus vessels. Third, the information on the utilization of lipid-lowering medications was not collected during the study, making it impossible to exclude their impact on the results. The TNT trial found that statin therapy reduced RC levels and cardiovascular risk in individuals with clinically evident CVDs [47]. In the FinnDiane study, the proportion of participants receiving lipid-lowering medications increased with increasing quartiles of RC, which indicated that, as discussed by the authors, the association between RC and outcome may be weakened by the efficacy of lipid-lowering drugs [15].

## Conclusions

This study found that RC may be a risk factor for DR among people who have had T2DM for a longer period of time, implying a probable time-accumulative effect on DR. Concomitantly, higher RC levels were correlated with wider retinal arterioles and venules and higher D_f_ values. Elevated RC levels could promote the onset and development of DR through the dilation of retinal venules. Therefore, individuals with long-standing T2DM must diligently monitor indicators of lipid metabolism. Controlling lipid levels is useful for mitigating the risk of microvascular complications. Additionally, the measurement and interpretation of retinal vascular parameters may be an effective combined preventive measure, especially for patients who do not show preclinical signs in fundus photographs but still possess risk factors (e.g., higher RC levels) for DR. More prospective studies are warranted to clarify whether RC can effectively predict DR, which is promising for opening new avenues for complication prevention.

### Supplementary Information

**Additional file 1: Fig. S1 **Subgroup analyses for correlation between RC and CRAE. **Fig. S2 **Subgroup analyses for correlation between RC and CRVE. **Fig. S3 **Subgroup analyses for correlation between RC and D_f_. **Table S1. **Binary logistic regression analyses for correlation between retinal vasculature and DR.

## Data Availability

The data that support the findings of this study are available from Shanghai Municipal Centers for Disease Control and Prevention but restrictions apply to the availability of these data, which were used under license for the current study, and so are not publicly available.

## Abbreviations

AVR: Arteriole to venule ratio
BMI: Body mass index
CI: Confidence interval
CRAE: Central retinal artery equivalent
CRVE: Central retinal vein equivalent
CVD: Cardiovascular disease
DBP: Diastolic blood pressure
D_f_: Fractal dimension
DR: Diabetic retinopathy
FPG: Fasting plasma glucose
HbA1c: Glycated hemoglobin A1c
HDL-C: High-density lipoprotein cholesterol
LDL-C: Low-density lipoprotein cholesterol
MAP: Mean arterial pressure
NPDR: Non-proliferative diabetic retinopathy
PD: Papillary diameter
PDR: Proliferative diabetic retinopathy
RC: Remnant cholesterol
Ref.: Reference
SBP: Systolic blood pressure
SCODE: Shanghai Cohort Study of Diabetic Eye Disease
T1DM: Type 1 diabetes mellitus
T2DM: Type 2 diabetes mellitus
TC: Total cholesterol
TG: Triglycerides
TRL: Triglyceride-rich lipoprotein
VT: Vascular tortuosity
