# Supplementary material for: Remnant cholesterol is correlated with retinal vascular morphology and diabetic retinopathy in type 2 diabetes mellitus: a cross-sectional study
Source: Lipids Health Dis. 2024 Mar 11;23:75. doi: 10.1186/s12944-024-02064-6 (PMC10926603; doi:10.1186/s12944-024-02064-6)
Supplement: Supplementary file 1 — Additional file 1: Fig. S1 Subgroup analyses for correlation between RC and CRAE. Fig. S2 Subgroup analyses for correlation between RC and CRVE. Fig. S3 Subgroup analyses for correlation between RC and Df. Table S1. Binary logistic regression analyses for correlation between retinal vasculature and DR. [file 12944_2024_2064_MOESM1_ESM.docx]

***Supplemental Materials***





**Fig. S1** Subgroup analyses for correlation between RC and CRAE. The stratification was based on the median of the age, T2DM duration, BMI, HbA1c, and MAP. **P*-trend < 0.05, ***P*-trend < 0.01, ****P*-trend < 0.001.





**Fig. S2** Subgroup analyses for correlation between RC and CRVE. The stratification was based on the median of the age, T2DM duration, BMI, HbA1c, and MAP. ***P*-trend < 0.01, ****P*-trend < 0.001.

**

**

**Fig. S3** Subgroup analyses for correlation between RC and D_f_. The stratification was based on the median of the age, T2DM duration, BMI, HbA1c, and MAP. ***P*-trend < 0.01, ****P*-trend < 0.001.

**Table S1** Binary logistic regression analyses for correlation between retinal vasculature and DR

| **Variable** | **Crude model** | **Model 1** | **Model 2** | **Model 3** |
| --- | --- | --- | --- | --- |
| CRAE | 1.005 (1.001, 1.009) ** | 1.006 (1.002, 1.009) ** | 1.006 (1.002, 1.010) ** | 1.006 (1.002, 1.010) ** |
| CRVE | 1.010 (1.008, 1.013) *** | 1.011 (1.008, 1.013) *** | 1.011 (1.008, 1.013) *** | 1.011 (1.008, 1.013) *** |
| AVR | 0.044 (0.016, 0.122) *** | 0.043 (0.015, 0.119) *** | 0.046 (0.016, 0.129) *** | 0.047 (0.017, 0.134) *** |
| VT, ×10^-4^ | 1.054 (1.015, 1.095) ** | 1.054 (1.015, 1.094) ** | 1.055 (1.016, 1.096) ** | 1.057 (1.017, 1.098) ** |
| D_f_ | 0.097 (0.027, 0.345) *** | 0.104 (0.026, 0.408) ** | 0.150 (0.037, 0.604) ** | 0.161 (0.040, 0.650) * |

Data were presented as OR (95% CI). **P* < 0.05. ***P* < 0.01. **P* < 0.001.

Crude model: unadjusted.

Model 1: age and gender.

Model 2: Model 1 + BMI, T2DM duration, HbA1c, and MAP.

Model 3: Model 2 + TG, HDL-C, and LDL-C.
